# Frequency of HIV-testing and factors associated with multiple lifetime HIV-testing among a rural population of Zambian men

**DOI:** 10.1186/s12889-015-2259-3

**Published:** 2015-09-24

**Authors:** B. Hensen, JJ Lewis, A. Schaap, M. Tembo, M. Vera-Hernández, W. Mutale, HA Weiss, J. Hargreaves, JSA Stringer, H. Ayles

**Affiliations:** Department of Social and Environmental Health Research, Faculty of Public Health and Policy, London School of Hygiene and Tropical Medicine, 15-17 Tavistock Place, London, WC1H 9SH UK; MRC Tropical Epidemiology Group, Faculty of Epidemiology and Population Health, London School of Hygiene and Tropical Medicine, London, UK; ZAMBART Project, Ridgeway Campus, University of Zambia, Nationalist Road, Lusaka, Zambia; University College London and Institute for Fiscal Studies, London, UK; Department of Public Health, University of Zambia School of Medicine, Lusaka, Zambia; Global Women’s Health Division, Department of Obstetrics & Gynecology; Institute for Global Health and Infectious Diseases, School of Medicine, University of North Carolina, Chapel Hill, North Carolina USA; Department of Clinical Research, Faculty of Infectious and Tropical Diseases, London School of Hygiene and Tropical Medicine, London, UK

## Abstract

**Background:**

Across sub-Saharan Africa, men's levels of HIV-testing remain inadequate relative to women’s. Men are less likely to access anti-retroviral therapy and experience higher levels of morbidity and mortality once initiated on treatment. More frequent HIV-testing by men at continued risk of HIV-infection is required to facilitate earlier diagnosis. This study explored the frequency of HIV-testing among a rural population of men and the factors associated with more frequent HIV-testing.

**Methods:**

We conducted a secondary analysis of a population-based survey in three rural district in Zambia, from February-November, 2013. Households (*N* = 300) in randomly selected squares from 42 study sites, defined as a health facility and its catchment area, were invited to participate. Individuals in eligible households were invited to complete questionnaires regarding demographics and HIV-testing behaviours. Men were defined as multiple HIV-testers if they reported more than one lifetime test. Upon questionnaire completion, individuals were offered rapid home-based HIV-testing.

**Results:**

Of the 2376 men, more than half (61 %) reported having ever-tested for HIV. The median number of lifetime tests was 2 (interquartile range = 1-3). Just over half (*n* = 834; 57 %) of ever-testers were defined as multiple-testers. Relative to never-testers, multiple-testers had higher levels of education and were more likely to report an occupation. Among the 719 men linked to a spouse, multiple-testing was higher among men whose spouse reported ever-testing (adjusted prevalence ratio = 3.02 95 % CI: 1.37-4.66). Multiple-testing was higher in study sites where anti-retroviral therapy was available at the health facility on the day of a health facility audit. Among ever-testers, education and occupation were positively associated with multiple-testing relative to reporting one lifetime HIV-test. Almost half (49 %) of ever-testers accepted the offer of home-based HIV-testing.

**Discussion:**

Reported HIV-testing increased among this population of men since a 2011/12 survey. Yet, only 35 % of all men reported multiple lifetime HIV-tests. The factors associated with multiple HIV-testing were similar to factors associated with ever-testing for HIV. Men living with HIV were less likely to report multiple HIV-tests and employment and education were associated with multiple-testing. The offer of home-based HIV-testing increased the frequency of HIV-testing among men.

**Conclusion:**

Although men's levels of ever-testing for HIV have increased, strategies need to increase the lifetime frequency of HIV-testing among men at continued risk of HIV-infection.

## Background

Annual HIV-testing and counselling (HTC) in high prevalence settings is recommended for individuals at continued risk of HIV infection to support early detection of HIV-infection and initiation of anti-retroviral therapy (ART) [1]. Mathematical models suggest that the provision of “high-quality” HTC services to all individuals will increase the HIV-prevention impact of HTC service delivery [2]. In settings where annual HTC is recommended, including Zambia, men’s levels of ever HIV-testing remain lower than is needed to link men testing HIV-positive into care [3–5]. Encouraging men to increase their lifetime frequency of HIV-testing may prove challenging [6].

Studies exploring risk factors for HIV-testing in sub-Saharan Africa highlight that age [3, 7–10], employment [4, 11], education [8, 10, 12] and socio-economic position [4, 12], marital status [8, 10], having heard of ART [4], community-level employment and HIV-knowledge [13] are associated with men ever-testing. Whether these factors also encourage men to test more frequently deserves exploration, to determine whether the expansion of HTC services has increased the frequency of HTC among men at risk of HIV-infection. Such evidence would support the development of strategies to reach men in need of annual HIV-testing.

This study describes the frequency of HIV-testing among a rural population of Zambian men and explores the factors associated with frequent HIV-testing. We hypothesized that, relative to never-testers, the factors associated with multiple HIV-testing would be similar to ever-testing for HIV. Among men with a history of ever HIV-testing, we hypothesized that men reporting frequent HIV-testing would differ in socio-demographic characteristics from men reporting one lifetime HIV-test. We also explore whether an offer of home-based HIV-testing in a research setting increases the frequency of testing among men with a history of HIV-testing.

## Methods

We analyzed data collected for a stepped-wedge cluster randomized trial (CRT): the Better Health Outcomes through Mentoring and Assessment (BHOMA) trial, which aims to strengthen the healthcare system [14]. Details of the intervention are published elsewhere [14, 15]. Briefly, BHOMA was implemented in 42 clusters, defined as a health facility and its catchment area, in three districts in Lusaka Province, Zambia. BHOMA aims to reduce age-adjusted all-cause and under-5 mortality, and is being evaluated through three rounds of household surveys [14]. Increasing HIV-testing is not a primary or secondary objective. However, health facilities were equipped with diagnostics and essential medicines [14], healthcare workers provided with protocols to guide adult visits alongside recruitment of community health workers to increase demand for health services [14]. The majority of BHOMA study sites were rural (*n* = 34, 81 %). Data for the present analysis were from the mid-line evaluation (February-November, 2013) after intervention implementation in all sites.

In each cluster, squares of 900 m2 were marked within a 3.8 km of the health facility [3, 14]. Computer-generated randomization was used to determine which squares would be visited and the order of visitation. All households in randomly selected squares where the survey was started were visited until 300 households were enumerated in each cluster.

### Data collection

Data collection tools included: household enumeration, and household and individual questionnaires. Due to financial constraints, households were either invited to complete a partial (household enumeration and questionnaire only) or full survey (household members asked to complete an individual questionnaire and offered measurements including blood glucose and pressure, and HIV-testing). Systematic random sampling was used to select households for participation in the full survey, with every 2.5th household offered the full survey (*n* = 6788; 57 %). Personal digital assistants (PDAs) informed research assistants which survey to offer a household prior to visitation. Data to estimate BHOMA’s primary outcome were obtained from household enumeration Repeat visits were only conducted if entire households were absent. Questionnaires were adapted from Demographic and Health Surveys (DHS) and administered using PDAs. Household questionnaires included questions on asset ownership and housing material. Individuals aged 15–59 years were eligible for the individual questionnaire. After questionnaire completion, individuals were offered voluntary HIV-testing (Determine™ HIV-1/2).

### Statistical analysis

We restricted analyses to men. Outcomes of interest included i) never-testing, ii) ever-testing (defined as testing and receiving the result of an HIV-test), and iii) multiple-testing (defined as reporting >1 lifetime HIV-test). Ever-testers reporting one lifetime HIV-test were defined as one-time testers. Men self-reporting that they were living with HIV were defined as multiple-testers if their first HIV-test was before the test in which they received their HIV-positive diagnosis.

We described never- and ever-testing among men with complete data on variables of interest: age, religion, marital status, occupation, education, head of household, history of TB-treatment, ever HIV-tested and household socioeconomic position (SEP). Among ever-testers, we described the proportion reporting one and multiple HIV-tests. We described acceptance of home-based HIV-testing.

We described the distribution of the outcomes by socio-demographic characteristics, temporary migrancy (defined as being absent ≥1 month in the 6 months preceding the survey), and a history of TB-treatment. During household enumeration, females were asked what the number of their spouse was as listed on the enumeration form. Using this number, females were linked to their spouse. For men linked to a spouse who completed a questionnaire, we described outcomes by whether the spouse was pregnant, reported having children or ever HIV-tested. At cluster-level, we described outcomes by ART availability at the local health facility, HIV-prevalence, whether or not ≥50 % of men reported employment and whether 25 % of men listed 3+ ways to prevent HIV-infection.

Data on whether unexpired ART was available at health facilities was obtained from a health facility audit (conducted in 2012) [16]. A household SEP indicator was developed using principal components analysis (PCA) [3]. PCA was conducted on households with no missing data, regardless of whether households completed the full or partial survey, whether an eligible man was present and without taking account of district or rural/urban residence. SEP scores were divided into quintiles.

**Fig. 1:**
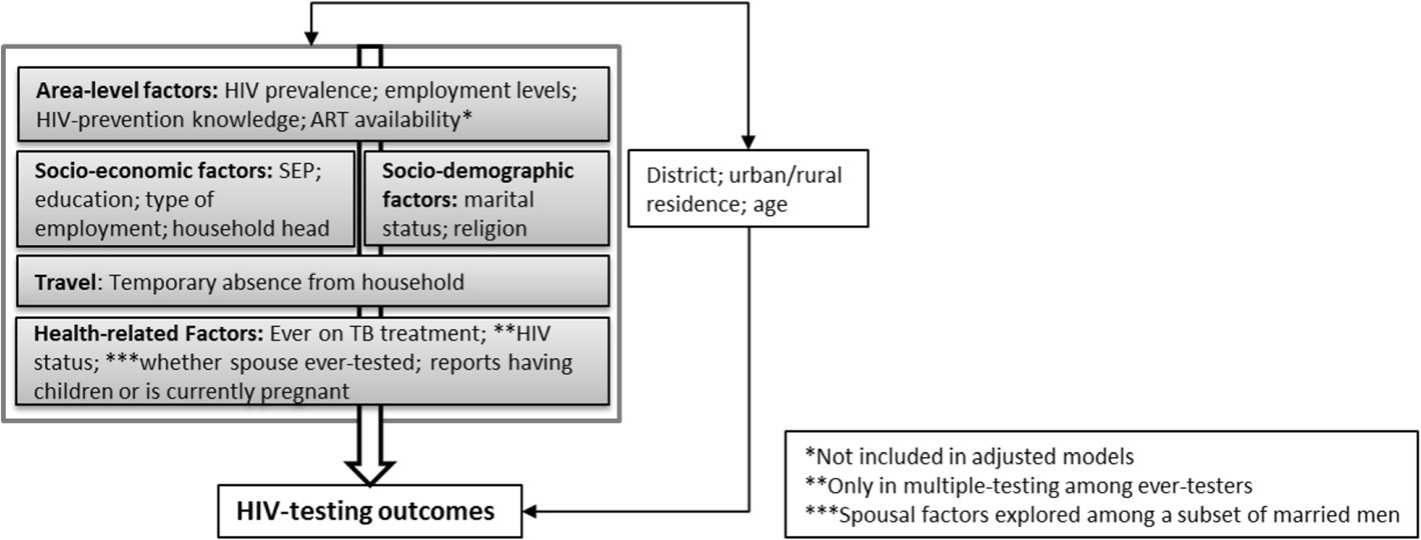
Framework illustrating the expected causal relationship between independent variables and HIV-testing

We estimated minimally-adjusted associations between independent variables and outcomes controlling for age, urban residence and a fixed effect for the three districts. Factors significant at the *p* ≤ 0.1 level in minimally-adjusted models were included in multivariable models based on the framework in Fig. 1. Socio-demographic factors were not adjusted for the more proximal factors likely to mediate their effect. Associations with community-level characteristics were estimated without adjustment for individual-level variables. Spousal characteristics were explored among the sub-set of men linked to a spouse. Multivariable models included a continuous variable for cluster size.

We fit random effects logistic regression models in Stata 13.0 to adjust for geographic clustering. We checked the reliability of model estimates by running the *quadchk* command. For age, education and SEP we conducted a test assuming linear trend if there appeared to be a linear association. Due to the high prevalence of the outcomes, we estimated associations with prevalence ratios (PRs) using marginal standardization to estimate PRs, and the delta method to estimate 95 % confidence intervals (95 % CI). We used the likelihood ratio test (LRT) to estimate p-values.

### Missing data

Survey non-participation was high due to men being absent at the time of the household survey. We used Heckman-type selection models to investigate the null hypothesis that outcomes were “missing at random” conditional on covariates available for non-participants [17–19]. We identified three variables that we theorized would be associated with survey participation but not HIV-testing: time (morning, afternoon, evening), day (Monday-Thursday, Friday, Saturday-Sunday) and season (rainy, cool/dry, hot/dry) of the survey. These variables were included in a random effects model controlling for variables crudely associated with participation to investigate whether they were independently associated with participation (Appendix 1: Table 4) [18]. Characteristics of eligible participants were randomly distributed by time but not day of the survey (Appendix 3: Table 6). Time was entered in the model as a selection variable. Data available on non-participants and associated with HIV-testing in a 2011/12 survey [3] or theorized to be associated were included in the outcome model. We assessed evidence for the null hypothesis using rho and its p-value [18]. Estimates of association between independent variables and outcome were obtained by adjusting for variables as described in Fig. 1. Cluster-level variables were not adjusted for proximal factors. We investigated whether adjustment for variables included in the Heckman models affected estimates of association.

### Ethics statement

BHOMA was approved by the University of Zambia Bioethics Committee, the London School of Hygiene and Tropical Medicine Ethics Committee and the institutional review boards at the University of Alabama at Birmingham (Birmingham, AL, USA) and University of North Carolina (Chapel Hill, NC, USA) [14]. Individuals were informed of the study objectives and asked for written informed consent. Consent was obtained from a parent/guardian for individuals aged 15–17 years.

## Results

### Sample population

**Fig. 2:**
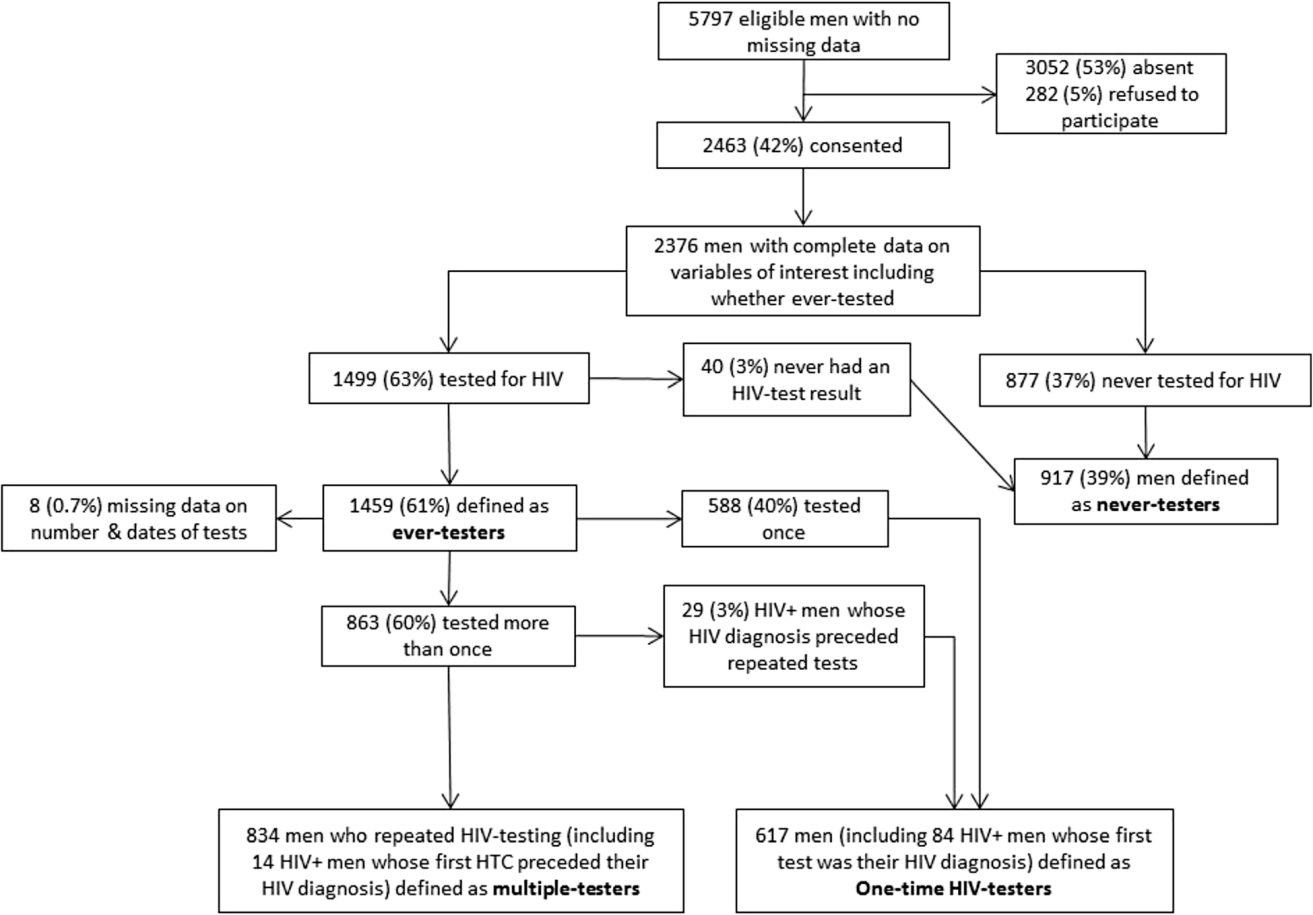
Flow diagram of study participation and frequency of HIV-testing

Of 5145 households invited to complete the full survey, 5144 consented. In these households, 6202 eligible men were enumerated of whom 29 did not have full data available and 376 were listed as absent in the month of or the month preceding the survey, leaving 5797 (93 %) men defined as eligible to participate. Among these men, 42 % (*n* = 2463) participated (Fig. 2). Participation ranged from 22–65 % (median: 42 %; inter-quartile range (IQR): 34–51 %) across study sites. Men of highest SEP were less likely to participate than men of lowest SEP (PR = 0.74, 95 % CI: 0.66-0.83; Appendix 1: Table 4). Men listed as a household head were more likely to participate (PR = 1.34 95 % CI: 1.25-1.43).

### Frequency of HIV-testing

Among participating men, 37 % (*n* = 877/2376) reported never-testing for HIV, and 2 % (*n* = 40/2376) tested but never received the result of an HIV-test. Overall, 61 % (*n* = 1459) of men ever-tested. Ever-testing ranged from 44–87 % (median = 62 %; IQR = 56–67 %) across study sites. Among ever-testers, 7 % (*n* = 98) reported themselves HIV-positive. The number of lifetime HIV-tests ranged from 1–25 (median = 2; IQR = 1–3).

**Fig. 3:**
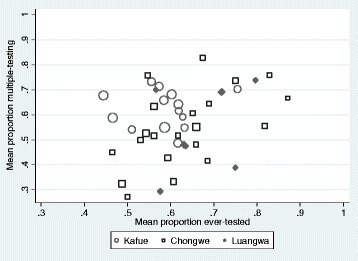
Scatter plot of correlation between multiple-testing among ever-testers and ever-testing at cluster-level

Just over half of ever-testers (57 %; *n* = 834/1459) were defined as multiple-testers (Fig. 2). Among ever-testers, levels of multiple-testing were 62 % in Kafue district and 55 % in Chongwe and Luangwa. There was evidence for correlation between multiple-testing among ever-testers and ever-testing in Chongwe district (Chongwe *r* = 0.54; *p* = 0.01; Kafue *r* = 0.05; *p* = 0.86; Luangwa *r* = 0.34; *p* = 0.46; Fig. 3). Multiple-testing ranged from 27– 83 % (median = 57 %; IQR: 48–68 %) across study sites and was clustered by study site (intra-cluster correlation coefficient (ICC) = 0.05 95 % CI: 0.03-0.11; *p* < 0.01). Just over half (57 %) the men living with HIV reported one-lifetime HIV-test. An estimated 14 % HIV-tested prior to the test in which they received an HIV-positive diagnosis.

The median numbers of years between first and most recent HIV-test was 2 (IQR: 1–4). Half (*n* = 422; 51 %) of multiple-testers and 29 % (*n* = 176) of one-time testers reported their first HIV-test between 2009 and 2011. Sixty-percent (*n* = 504) of multiple-testers and 31 % (*n* = 191) of one-time testers tested within 12 months of the survey. Over half of one-time (*n* = 341; 55 %) and multiple-testers (*n* = 498; 60 %) reported their most recent HIV-test at the local health facility.

### Factors associated with multiple HIV-testing

Relative to never-testers, multiple-testing was higher among men aged 30–39 relative to men 20–29 (65 % vs 53 %; adjPR = 1.25 95 % CI: 1.12-1.39; Table 1), men with complete secondary/higher education relative to men with no/primary education (65 % vs 43 %; adjPR = 1.59 95 % CI: 1.38-1.81) and among men reporting service/professional employment relative to men reporting no employment (70 % vs 33 %; adjPR = 1.29 95 % CI: 1.08-1.50; Table 1). Multiple-testing was higher among married/cohabiting men relative to single men (61 % vs 32 %; adjPR = 1.23 95 % CI: 1.03-1.43) and among Protestant men (52 %) relative to men of no religion (33 %; adjPR = 0.69 95 % CI: 0.47-0.90). There was weak evidence that men of middle SEP were more likely to report multiple-testing relative to men of lowest SEP (adjPR = 1.19 95 % CI: 1.02-1.37). Having a spouse who reported ever-testing was associated with multiple-testing (adjPR = 3.02 95 % CI: 1.37-4.66) with little evidence that having children was associated (*p* = 0.20). There was little evidence that multiple-testing differed by cluster-level employment or HIV-knowledge. Multiple-testing was higher in sites where ART was available on the day of the audit (52 % vs 43 %; adjPR = 1.29 95 % CI: 1.12-1.45).

**Table 1:** Distribution of characteristics by never- and multiple-testers and factors associated with multiple HIV-testing relative to never-testers (*N* = 1751)

	Details	Multiple-testers & never-testers (n, col %)	Never-Testers (n, row %)	Multiple Testers (n, row %)	Minimally-adjusted PR (95 % CI)^b^	Adjusted PR (95 % CI)^c^	*p*-value^d^
Age	15-19	387 (22.1)	328 (84.8)	59 (15.2)	0.28 (0.21-0.36)	0.29 (0.22-0.37)	<0.01
	20-29	553 (31.6)	259 (46.8)	294 (53.2)	1.0	1.0	
	30-39	391 (22.3)	137 (35.0)	254 (65.0)	1.23 (1.09-1.36)	1.25 (1.12-1.39)	(<0.01)
	≥40	420 (24.0)	193 (46.0)	227 (54.0)	1.02 (0.89-1.14)	1.04 (0.91-1.16)	
Head of household	No	680 (38.8)	472 (69.4)	208 (30.6)	1.0	1.0	0.06
	Yes	1071 (61.2)	445 (41.5)	626 (58.5)	1.28 (1.09-1.46)	1.15 (0.97-1.32)	
Religion	Protestant	684 (39.1)	332 (48.5)	352 (51.5)	1.0	1.0	0.01
	Catholic	435 (24.8)	229 (52.6)	206 (47.4)	0.89 (0.78-1.00)	0.91 (0.80-1.01)	
	SDA	257 (14.7)	122 (47.5)	135 (52.5)	1.01 (0.88-1.15)	0.98 (0.85-1.11)	
	Other	305 (17.4)	187 (61.3)	118 (38.7)	0.78 (0.66-0.90)	0.83 (0.71-0.96)	
	None	70 (4.0)	47 (67.1)	23 (32.9)	0.59 (0.38-0.79)	0.69 (0.47-0.90)	
Marital status	Single	762 (43.5)	522 (68.5)	240 (31.5)	1.0	1.0	0.02
	Married/cohabiting	913 (52.1)	361 (39.5)	552 (60.5)	1.33 (1.12-1.53)	1.23 (1.03-1.43)	
	Ever married	76 (4.3)	34 (44.7)	42 (55.3)	1.26 (0.94-1.59)	1.30 (0.99-1.61)	
Education	No/Primary	745 (42.5)	426 (57.2)	319 (42.8)	1.0	1.0	
	Incomplete secondary	649 (37.1)	366 (56.4)	283 (43.6)	1.29 (1.13-1.44)	1.26 (1.11-1.40)	<0.01
	Secondary or higher	357 (20.4)	125 (35.0)	232 (65.0)	1.61 (1.41-1.81)	1.59 (1.38-1.81)	(<0.01)
Occupation	None	802 (45.8)	536 (66.8)	266 (33.2)	1.0	1.0	<0.01
	Agriculture (others land)	378 (21.6)	182 (48.1)	196 (51.9)	1.17 (0.99-1.34)	1.11 (0.94-1.27)	
	Agriculture (own land)	357 (20.4)	134 (37.5)	223 (62.5)	1.39 (1.19-1.60)	1.30 (1.11-1.48)	
	Services/Professional	214 (12.2)	65 (30.4)	149 (69.6)	1.53 (1.30-1.77)	1.29 (1.08-1.50)	
SEP Group	Lowest	343 (19.6)	184 (53.6)	159 (46.4)	1.0	1.0	0.06
	Low	331 (18.9)	188 (56.8)	143 (43.2)	1.01 (0.83-1.18)	0.99 (0.93-1.14)	
	Middle	365 (20.8)	179 (49.0)	186 (51.0)	1.24 (1.03-1.44)	1.19 (1.02-1.37)	
	High	361 (20.6)	189 (52.4)	172 (47.6)	1.21 (1.01-1.42)	1.09 (0.92-1.26)	
	Highest	351 (20.0)	177 (50.4)	174 (49.6)	1.32 (1.08-1.55)	1.04 (0.84-1.25)	
Present continuously previous 6mths	No	87 (5.0)	50 (57.5)	37 (42.5)	1.0	-	-
	Yes	1664 (95.0)	867 (52.1)	797 (47.9)	1.12 (0.85-1.40)		
History of TB treatment	No	1702 (97.2)	899 (52.8)	803 (47.2)	1.0	-	-
	Yes	49 (2.8)	18 (36.7)	31 (63.3)	1.15 (0.88-1.43)	-	-
Spousal Characteristics (*N* = 511)							
Currently pregnant	No	447 (87.6)	179 (40.0)	268 (60.0)	1.0	-	-
	Yes	63 (12.4)	19 (30.2)	44 (69.8)	1.16 (0.95-1.38)		
Has Children	No	27 (5.3)	14 (51.9)	13 (48.2)	1.0	1.0	0.20
	Yes	483 (94.7)	183 (37.9)	300 (62.1)	1.34 (0.76-1.91)	1.26 (0.76-1.76)	
Wife previously HIV-tested	No	56 (11.0)	46 (82.1)	10 (17.9)	1.0	1.0	<0.01
	Yes	454 (89.0	151 (33.3)	303 (66.7)	3.53 (1.50-5.57)	3.02 (1.37-4.66)	
Cluster-level Characteristics							
>50 % of men employed	No	684 (39.1)	379 (55.4)	305 (44.6)	1.0	-	-
	Yes	1067 (60.9)	538 (50.4)	529 (49.6)	1.10 (0.91-1.29)	-	-
>25 % mention 3+ ways to prevent HIV infection	No	878 (50.1)	435 (49.5)	443 (50.5)	1.0	-	-
	Yes	873 (49.9)	482 (55.2)	391 (44.8)	0.90 (0.76-1.04)	-	-
HIV Prevalence	<10 %	1381 (78.9)	704 (51.0)	677 (49.0)	1.0	1.0	0.14
	10 %+	370 (21.1)	213 (57.6)	157 (42.4)	0.87 (0.70-1.03)	0.87 (0.70-1.04)	
ART Available at Health Facility^a^	No	858 (51.8)	491 (57.2)	367 (42.8)	1.0	1.0	<0.01
	Yes	799 (48.2)	381 (47.7)	418 (52.3)	1.30 (1.12-1.48)	1.29 (1.12-1.45)	

^a^94 missing data *N* = 1657; ^b^Adjusted for age, urban/rural residence and district; ^c^Adjusted for variables higher in the conceptual framework (Fig. 1); ^d^For adjusted model and based on LRT, *p*-value in brackets is assuming linear trend

^e^ever-married means either widowed, separated or divorced

Relative to one-time testers, multiple-testers were less likely to be aged 15–19 (adjPR compared to 20–29: 0.63 95 % CI: 0.49-0.77; Table 2). Among men working on their own land, 70 % reported multiple-testing relative to 48 % of men reporting no employment (adjPR = 1.45 95 % CI 1.27-1.63). There was little evidence of an association with marital status, a history of TB treatment or household SEP, with weak evidence that multiple-testing differed by being household head, pregnancy status of the spouse, or having children (Table 2). Men living with HIV were less likely to report multiple-tests prior to diagnosis (14 % vs 61 % among HIV-negative men; adjPR = 0.22; 95 % CI: 0.11-0.33). There was little evidence for an association with ART availability or cluster-level employment. Multiple-testing was lower in clusters with higher levels of HIV-prevention knowledge (53 % vs 62 %; adjPR =0.86 95 % CI: 0.74-0.98).

**Table 2:** Distribution of characteristics by one- and multiple-testers and factors associated with multiple HIV-testing relative to one-time testers (*N* = 1451)

	Details	Men with a history of ever-testing	One-time Testers (n, row %)	Multiple Testers (n, row %)	Minimally-adjusted PR (95 % CI)^b^	Adjusted PR (95 % CI)^c^	*p*-value^d^
Age	15-19	159 (11.0)	100 (62.9)	59 (37.1)	0.62 (0.48-0.76)	0.63 (0.49-0.77)	<0.01 (0.03)
	20-29	489 (33.7)	195 (39.9)	294 (60.1)	1.0	1.0	
	30-39	405 (27.9)	151 (37.3)	254 (62.7)	1.06 (0.95-1.18)	1.05 (0.94-1.16)	
	≥40	398 (27.4)	171 (43.0)	227 (57.0)	0.95 (0.84-1.06)	0.95 (0.84-1.06)	
Head of household	No	424 (29.2)	216 (50.9)	208 (49.1)	1.0	1.0	0.06
	Yes	1027 (70.8)	401 (39.0)	626 (61.0)	1.15 (1.00-1.30)	1.13 (0.98-1.28)	
Religion	Protestant	575 (39.6)	223 (38.8)	352 (61.2)	1.0	1.0	0.15
	Catholic	386 (26.6)	180 (46.6)	206 (53.4)	0.87 (0.77-0.98)	0.89 (0.79-0.99)	
	SDA	235 (16.2)	100 (42.6)	135 (57.4)	0.90 (0.78-1.03)	0.89 (0.77-1.01)	
	Other	208 (14.3)	90 (43.3)	118 (56.7)	0.92 (0.79-1.05)	0.99 (0.86-1.11)	
	None	47 (3.2)	24 (51.1)	23 (48.9)	0.78 (0.54-1.02)	0.86 (0.62-1.09)	
Marital status	Single	475 (32.7)	235 (49.5)	240 (50.5)	1.0	-	-
	Married/cohabiting	905 (62.4)	353 (39.0)	552 (61.0)	1.15 (0.99-1.31)		
	Ever married	71 (4.9)	29 (40.8)	42 (59.2)	1.13 (0.87-1.40)		
Education	No/Primary	589 (40.6)	270 (45.8)	319 (54.2)	1.0	1.0	
	Incomplete secondary	515 (35.5)	232 (45.0)	283 (55.0)	1.08 (0.96-1.21)	1.11 (0.99-1.24)	<0.01 (<0.01)
	Secondary or higher	347 (23.9)	115 (33.1)	232 (66.9)	1.26 (1.11-1.41)	1.29 (1.13-1.44)	
Occupation	None	555 (38.2)	289 (52.1)	266 (47.9)	1.0	1.0	<0.01
	Agriculture (others land)	351 (24.2)	155 (44.2)	196 (55.8)	1.11 (0.96-1.27)	1.10 (0.95-1.26)	
	Agriculture (own land)	320 (22.1)	97 (30.3)	223 (69.7)	1.46 (1.28-1.65)	1.45 (1.27-1.63)	
	Services/Professional	225 (15.5)	76 (33.8)	149 (66.2)	1.27 (1.09-1.46)	1.19 (1.00-1.37)	
SEP Group	Lowest	263 (18.1)	104 (39.5)	159 (60.5)	1.0	-	-
	Low	264 (18.2)	121 (45.8)	143 (54.2)	0.91 (0.77-1.04)		
	Middle	336 (23.2)	150 (44.6)	186 (55.4)	0.90 (0.77-1.04)		
	High	311 (21.4)	139 (44.7)	172 (55.3)	0.92 (0.78-1.06)		
	Highest	277 (19.1)	103 (37.2)	174 (62.8)	1.05 (0.88-1.21)		
Present continuously previous 6mths	No	60 (4.1)	23 (38.3)	37 (61.7)	1.0	-	-
	Yes	1391 (95.9)	594 (42.7)	797 (57.3)	0.91 (0.72-1.09)		
History of TB treatment	No	1383 (95.3)	580 (41.9)	803 (58.1)	1.0	1.0	0.62
	Yes	68 (4.7)	37 (54.4)	31 (45.6)	0.76 (0.55-0.97)	1.06 (0.84-1.28)	
HIV Status	Negative	1297 (93.0)	507 (39.1)	790 (60.9)	1.0	1.0	<0.01
	Positive	98 (7.0)	84 (85.7)	14 (14.3)	0.20 (0.10-0.31)	0.22 (0.11-0.33)	
Spousal Characteristics (*N* = 517)							
Currently pregnant	No	443 (85.7)	175 (39.5)	268 (60.5)	1.0	--	--
	Yes	74 (14.3)	30 (40.5)	44 (59.5)	0.97 (0.77-1.17)		
Has Children	No	No	20 (3.9)	13 (65.0)	1.0	-	-
	Yes	Yes	497 (96.1)	299 (60.2)	0.93 (0.60-1.26)		
Wife previously HIV-tested	No	24 (4.6)	14 (58.3)	10 (41.7)	1.0	1.0	0.32
	Yes	493 (95.4)	191 (38.7)	303 (61.3)	1.44 (0.75-2.12)	1.19 (0.73-1.66)	
Cluster-level Characteristics							
>50 % of men employed	No	578 (39.8)	273 (47.2)	305 (52.8)	1.0	1.0	0.29
	Yes	873 (60.2)	344 (39.4)	529 (60.6)	1.15 (0.97-1.34)	1.09 (0.92-1.25)	
>25 % mention 3+ ways to prevent HIV infection	No	711 (49.0)	268 (37.7)	443 (62.3)	1.0	1.0	0.05
	Yes	740 (51.0)	349 (47.2)	391 (52.8)	0.84 (0.72-0.95)	0.86 (0.74-0.98)	
HIV Prevalence	<10 %	1160 (79.9)	483 (41.6)	677 (58.4)	1.0	-	-
	10 %+	291 (20.1)	134 (46.0)	157 (54.0)	0.96 (0.79-1.13)		
ART Available at Health Facility^a^	No	676 (49.3)	309 (45.7)	367 (54.3)	1.0	-	-
	Yes	694 (50.7)	276 (39.8)	418 (60.2)	1.09 (0.95-1.24)		

^a^81 missing data *N* = 1370; ^b^Adjusted for age, urban/rural residence and district; ^c^Adjusted for variables higher in the conceptual framework (Fig. 1); ^d^For adjusted model and based on LRT, *p*-value in brackets is assuming linear trend

^e^N=1395 as 56 men were missing data on self-reported HIV status

### Acceptance of an offer of home-based HIV-testing

Almost half of never- and ever-testers accepted the offer of home-based HIV-testing (48 %; *n* = 449 & 49 %; *n* = 719, respectively). Acceptance among ever-testers was clustered by study site (median: 48.0 % IQR: 40.0-54.7 %; ICC = 0.06 95 % CI 0.03-0.11; *p* < 0.01). Acceptance was similar among multiple- (*n* = 422; 51 %) and one-time testers (*n* = 292; 47 %; adjPR = 1.05 95 % CI: 0.93-1.17; Table 3). Among men reporting themselves HIV-negative or who did not know their HIV-status, 3 % tested HIV-positive at this test.

**Table 3:** Acceptance of an offer of home-based HIV-testing by socio-demographic characteristics and factors associated with acceptance among ever-testers (*N* = 1459)

	Details	Distribution (n, col %)	HBHTC (n, row %)	Minimally-Adjusted PR (95 % CI)^d^	Adjusted PR (95 % CI)^e^	*p*-value^f^
	Ever tested	*N* = 1459	719 (49.0)	-	-	-
Age	15-19	159 (10.9)	89 (56.0)	0.98 (0.82-1.14)	0.97 (0.81-1.14)	0.01 (<0.01)
	20–29	492 (33.7)	272 (55.6)	1.0	1.0	
	30–39	408 (28.0)	187 (46.2)	0.83 (0.72-0.95)	0.84 (0.73-0.95)	
	≥40	400 (27.4)	166 (41.7)	0.75 (0.65-0.86)	0.76 (0.65-0.87)	
Household Head	No	425 (29.1)	239 (56.2)	1.0	1.0	0.02
	Yes	1034 (70.9)	480 (46.4)	0.88 (0.76-1.00)	0.85 (0.74-0.97)	
Education	None/primary	591 (40.5)	285 (48.4)	1.0	1.0	0.32
	Some secondary	517 (35.4)	274 (53.2)	1.05 (0.92-1.18)	1.05 (0.92-1.19)	
	Complete secondary/higher	351 (24.1)	155 (44.7)	0.89 (0.76-1.03)	0.94 (0.79-1.10)	
Occupation	None	557 (38.2)	298 (53.7)	1.0	-	-
	Agriculture (others land)	352 (24.1)	170 (48.4)	0.98 (0.84-1.12)		
	Agriculture (own land)	322 (22.1)	150 (46.9)	0.95 (0.80-1.10)		
	Services/Professional	228 (15.6)	96 (42.7)	0.85 (0.70-1.00)		
Religion	Protestant	577 (40.0)	267 (46.4)	1.0	-	-
	Catholic	390 (26.7)	204 (52.8)	1.06 (0.92-1.20)		
	SDA	235 (16.1)	107 (45.5)	0.96 (0.80-1.12)		
	Other	210 (14.4)	112 (53.8)	1.08 (0.91-1.25)		
	None	47 (3.2)	24 (51.1)	1.03 (0.71-1.34)		
Marital status	Single	477 (32.7)	259 (54.5)	1.0	-	-
	Married/cohabiting	910 (62.4)	419 (46.3)	0.96 (0.81-1.10)		
	Ever married	72 (4.9)	36 (50.7)	1.12 (0.85-1.40)		
Household SEP Group	Lowest	264 (18.1)	143 (54.4)	1.0	1.0	0.29 (0.13)
	Low	265 (18.2)	137 (51.9)	1.01 (0.84-1.18)	1.00 (0.83-1.18)	
	Middle	337 (23.1)	172 (51.2)	1.02 (0.85-1.18)	1.01 (0.84-1.17)	
	High	313 (21.5)	150 (48.2)	0.98 (0.81-1.15)	0.97 (0.80-1.14)	
	Highest	280 (19.2)	112 (40.4)	0.81 (0.64-0.99)	0.82 (0.64-1.01)	
Present continuously in previous 6mths	No	60 (4.1)	38 (63.3)	1.0	1.0	0.04
	Yes	1399 (95.9)	676 (48.6)	0.79 (0.62-0.96)	0.79 (0.62-0.95)	
History of TB treatment	No	1391 (95.3)	689 (49.8)	1.0	1.0	0.71
	Yes	68 (4.7)	25 (36.8)	0.80 (0.56-1.04)	0.95 (0.68-1.21)	
HIV status^a^	Negative	1305 (93.0)	675 (51.7)	1.0	1.0	<0.01
	Positive	98 (7.0)	21 (21.4)	0.47 (0.29-0.64)	0.49 (0.30-0.68)	
History of multiple HIV-testing^b^	No	617 (42.5)	292 (47.3)	1.0	1.0	0.41
	Yes	834 (57.5)	422 (50.6)	1.10 (0.98-1.22)	1.05 (0.93-1.17)	
Spouse Characteristics (*N* = 520)						
Currently pregnant	No	446 (85.8)	231 (51.8)	1.0	1.0	0.12
	Yes	74 (14.2)	30 (40.5)	0.78 (0.56-1.00)	0.82 (0.60-1.03)	
Wife Reports having ≥1 Child	No	21 (4.0)	10 (47.6)	1.0	-	-
	Yes	499 (96.0)	252 (50.4)	1.10 (0.58-1.62)		
Wife previously HIV-tested	No	24 (4.6)	17 (70.8)	1.0	1.0	0.01
	Yes	496 (95.4)	245 (49.3)	0.67 (0.50-0.84)	0.66 (0.50-0.81)	
Cluster-level Factors						
>50 % of men employed	No	581 (39.8)	326 (56.4)	1.0	1.0	0.30
	Yes	878 (60.2)	388 (44.4)	0.88 (0.75-1.01)	0.92 (0.78-1.06)	
>25 % mention 3+ ways to prevent HIV infection	No	715 (49.0)	307 (43.2)	1.0	1.0	0.13
	Yes	744 (51.0)	407 (55.0)	1.16 (0.99-1.32)	1.12 (0.95-1.30)	
HIV Prevalence	<10 %	1165 (79.8)	576 (49.7)	1.0	-	-
	>10 %	294 (20.2)	138 (47.4)	0.98 (0.81-1.16)		
ART Available at Health Facility^c^	No	680 (49.4)	338 (50.0)	1.0	-	-
	Yes	697 (50.6)	328 (47.3)	0.97 (0.82-1.12)		

Number missing data: ^b^8 men missing data on dates of first and last test N=1451 a 56 missing HIV status data; *N* = 1395; ^c^82 missing data; ^d^Adjusted for age, urban/rural residence and district; ^e^Adjusted for variables higher in the conceptual framework (Fig. 1); ^f^For adjusted model & based on LRT, *p*-value in brackets is for assuming linear trend

Acceptance was lower among men aged ≥40 years relative to men aged 20–29 (42 % vs 56 %; adjPR = 0.76; 95 % CI: 0.65-0.87). There was little evidence that acceptance was associated with occupation, education, religion or marital status. Men present continuously in the 6 months preceding the survey were less likely to accept the offer relative to men with a period of being absent (adjPR = 0.79 95 % CI: 0.62-0.95) as were men whose spouse ever-tested (adjPR = 0.66 95 % CI: 0.50-0.81). Acceptance was lower among men listed as a household head (adjPR relative to men not a head = 0.85 95 % CI: 0.74-0.97) and among men of highest SEP (40 %) relative to men of lowest SEP (54 %; adjPR = 0.82 95 % CI: 0.64-1.01) with some evidence for a linear trend with SEP (*p* = 0.14). There was little evidence that cluster-level employment, HIV-prevalence or ART availability were associated with acceptance. There was weak evidence that acceptance was higher in clusters with higher HIV-prevention knowledge (55 % vs 43 %; adjPR = 1.12 95 % CI: 0.95-1.30).

### Heckman-type selection modelling

Participation was somewhat higher among men visited on Saturday/Sunday (48 %) relative to Monday-Thursday (41 %) and among men visited in the afternoon (45 %) relative to the morning (41 %; adjPR = 1.08 95 % CI 1.01-1.14; (Appendix 2: Table 5)). There was little evidence for unobserved factors influencing survey participation and HIV-testing outcomes (ever-testing: rho = −0.12 95 % CI:-0.93 to 0.88; *p* = 0.88; multiple-testing: rho = 0.20 95 % CI:-0.87 to 0.94; *p* = 0.80) however, confidence intervals were very wide. Results were similar when day of the week was included in the selection models Estimates of association between independent variables and multiple-testing were similar when adjusting for variables included in the Heckman-type selection model.

## Discussion

In this large, population-based survey of predominantly rural men, 61 % (95 % CI: 58-64 %) of men were defined as ever-testers. Over half the men with a history of HIV-testing reported more than one lifetime HIV-test. Factors associated with multiple-testing were similar to factors associated with ever-testing [3, 8, 10, 12]. The offer of home-based HIV-testing increased the lifetime frequency of HIV-testing among half of one-time and never-testers.

Limitations of this study are that, as data were cross-sectional, temporal relationships cannot be inferred. Data were self-reported and collected retrospectively. Men may have over-reported HIV-testing and there are likely to be errors in recalling dates of HIV-tests. As a secondary analysis of data collected for an unrelated CRT, the study had limited capacity to explore whether men were HIV-testing annually as data were collected on years since first and most recent test, and number of HIV-tests. Nonetheless, most multiple-testers first tested in 2009 or later, suggesting that recent expansions of HTC services, including PITC, have increased men’s frequency of HIV-testing. In the absence of data on sexual behaviours we had limited ability to explore whether multiple-testers were at increased risk of HIV-infection. However, we found that few men living with HIV reported HIV-testing prior to diagnosis. Although this measure is subject to limitations as multiple-testing was inferred from date of first HIV-test and of HIV-diagnosis, with almost 60 % of HIV-positive men reporting one lifetime test, findings suggest that a high proportion of men continue to be diagnosed on their first HIV-test. Further exploration of multiple-testing behaviours alongside data on sexual behaviours is needed. Some 60 % of married men were linked to their spouse; associations with spousal characteristics may be biased if characteristics of spouses linked differed from those not linked. Generalisability may be limited as the health system strengthening intervention, implemented in all sites at the time of data collection, may have contributed to increased frequency of HIV-testing.

Finally, outcomes were at risk of bias due to non-participation. Studies have shown that Heckman-type selection models can be used to correct HIV-prevalence estimates where refusal to HIV-test is high [18, 20]. We used Heckman-models as we theorised that non-participation, largely due to absence, may be related to HIV-testing behaviours. The models suggested that there was no evidence for unobserved factors associated with participation and HIV-testing outcomes. However, we had limited ability to model selection due to limited individual-level data on non-participants. The selection variables were weak predictors of participation and may not be valid exclusion restrictions [21], as survey timing within clusters was not randomly determined. Aspects of survey conduct may independently affect outcomes [18]; hence our estimates of correlation (rho) between outcome and participation had little precision.

Despite limitations, this study includes a large population of rural Zambian men whose multiple-testing behaviours have been understudied to date. The study provides important insights into the contribution of expanded HIV-testing service delivery to increasing men’s lifetime frequency of HIV-testing.

To our knowledge, there are relatively few population-based surveys exploring the factors associated with multiple-testing. In a 2007 population-based survey conducted in communities in Soweto, South Africa, 50 % of male ever-testers reported more than one lifetime test [4]. Multiple-testing was higher among individuals who had heard of ART [4]. In our study, multiple-testing was higher in clusters where ART was available at the health facility suggesting that expanded ART availability contributes not only to ever-testing [22], but to increased frequency of HIV-testing. In South and Central Province, Zambia (2010/11), 36 % of men ever-tested among whom 50 % reported >1 lifetime HIV-test [23]. In a 2012 nationally representative survey, 63 % of Kenyan males aged 15–64 years ever-tested with a median of 3 tests (IQR: 2–4) per person [10].

By the time of this study, HIV-testing services had been expanded across Zambia, couples HTC was recommended in ANC [24] and there was increased service promotion. Men whose spouse ever-tested were more likely to report multiple-tests. Similar to other settings, these findings suggest that HTC in ANC has provided men with access to HTC and may provide frequent access to HTC [12, 25]. Yet, few men attend ANC [25]. Considering the risk of HIV transmission among sero-discordant cohabiting/married couples, there remains a need to strengthen the delivery of HTC services to men through ANC [26–28].

Similar to a survey in South Africa, multiple-testers in this study were more likely to have complete secondary/higher education [4]. Employed men in this study were more likely to report multiple HIV-tests unlike in South Africa [4]. Formal employment may provide access to HTC services through the workplace thereby removing opportunity and financial costs of accessing facility-based HTC [29]. Alternatively, employed men may be encouraged by their employer or motivated by their role as providers to access health services [30]. Men of lower socioeconomic markers may face unique barriers to accessing HTC services that influence their frequency of HIV-testing. Lower health literacy likely contributes to lower levels of multiple-testing among men with less education. Other contributing factors, such as ability to access available services, stigma associated with HIV-testing within social networks or as experienced from healthcare workers, may also influence men’s frequency of HIV-testing [31]. Understanding why socioeconomic factors continue to influence men’s HIV-testing behaviors in the context of expanded service availability, the need for regular HIV-testing by socioeconomic factors and how to encourage men with lower levels of education or no formal employment to regularly test for HIV needs exploration.

Evidence suggests that men continue to access care at later stages of HIV-infection [32]. Regular-testing facilitates earlier diagnosis and opportunities to provide risk reduction counselling to HIV-negative individuals at higher risk of infection. In a facility-based cohort in South Africa, repeat-testers were less likely to be HIV-infected relative to first-time testers [33]. . In Uganda, South Africa and Zimbabwe, studies found that individuals at lower risk of HIV are more likely to ever- or repeat-test [4, 12, 34]. Conversely, in serological surveys in Tanzania, high-risk individuals were more likely to repeatedly accept VCT [35]. In this study, 40 % of ever-testers reported one-lifetime HIV-test and few men living with HIV tested before their diagnoses. With investment in delivering community-based HTC [36], there is a need to monitor whether those in greatest need of annual HIV-testing are accessing services and the effects of frequent HIV-testing on sexual behaviors [37]. Traditional “know your status” messaging may require reframing to emphasize the importance of annual HIV-testing if at ongoing risk of HIV-infection.

Home-based HIV-testing increased the lifetime frequency of HIV-testing among men in this study. As in other studies, there was little evidence that acceptance differed by markers of SEP [38, 39]. The relatively high refusal in our study relative to others [3, 40] likely reflects service delivery in the context of research, where the priority was data collection, rather than the acceptability of a home-based HTC programme [3]. In this study, multiple-testing was lower, but acceptance of home-based HIV-testing higher, in communities with higher HIV-prevention knowledge. These findings contribute to suggestions that poor accessibility influences men’s uptake of HTC services [38, 41]. Home-based HTC remains an important strategy to increase the frequency of HIV-testing among rural Zambian men with less access to services [3, 39]. However, with most men not home during household visits, a cost-effective strategy for offering regular home-based HIV-testing in rural settings requires exploration [40].

## Conclusion

Effective strategies to reach men with HTC services are available [28], and levels of ever-testing increased among this population of men [3]. However, only 35 % of all men reported multiple HIV-tests and few men living with HIV reported HIV-testing before being diagnosed. More effective implementation and delivery of available HTC services is required to reach men in need of frequent HTC [42]. Novel alternatives to encourage never-testers to access existing HTC services should be explored [28]. These strategies could include self-testing and incentivised testing, shown to be acceptable and feasible among men [43–45]. Additional research to investigate models for delivery, yield of these strategies and whether they are effective at increasing HIV-testing among HIV-negative men at high risk of infection is required [46, 47].

## Appendix

**Table 4:** Characteristics of eligible men & Factors Associated with Participation (*N* = 5797)

Characteristic	Detail	Distribution (n, col %)	Participants (n, row %)	Crude PR^a^ (95 % CI)
All men	5797	-	2463 (42.5)	-
Age category	15-24	2303 (39.7)	934 (40.6)	1.0
	25-60	3494 (60.3)	1529 (43.8)	1.08 (1.02-1.15)
Head of household	No	2590 (44.7)	921 (35.6)	1.0
	Yes	3207 (55.3)	1542 (48.1)	1.34 (1.25-1.43)
Present continuously previous 6mths	No	223 (3.8)	116 (52.0)	1.0
	Yes	5574 (96.2)	2347 (42.1)	0.80 (0.69-0.91)
SEP	Lowest	957 (16.5)	463 (43.4)	1.0
	Low	1038 (17.9)	471 (45.4)	0.96 (0.87-1.06)
	Middle	1206 (20.8)	536 (44.4)	0.93 (0.84-1.02)
	High	1275 (22.0)	517 (40.6)	0.84 (0.76-0.93)
	Highest	1321 (22.8)	476 (36.0)	0.74 (0.66-0.83)
Household size	1-5	2736 (47.2)	1325 (48.4)	1.0
	6–10	2711 (46.8)	1029 (38.0)	0.78 (0.73-0.83)
	>10	350 (6.0)	109 (31.1)	0.64 (0.53-0.75)
Urban cluster	No	4751 (82.0)	2042 (43.0)	1.0
	Yes	1046 (18.0)	421 (40.3)	0.94 (0.75-1.13)
District	Kafue	2106 (36.3)	886 (42.1)	1.0
	Chongwe	2810 (48.5)	1122 (39.9)	0.95 (0.81-1.10)
	Luangwa	881 (15.2)	455 (51.7)	1.23 (1.01-1.45)

**Table 5:** Distribution of eligible men by selection variables and association between participation and selection variables (*N* = 5797)

Selection Variables	Description	Distribution	Participants	Crude PR	Adjusted PR^a^	*p*-value^b^
Time of Survey	Morning (630–1159)	2778 (47.9)	1140 (41.0)	1.0	1.0	0.02
	Afternoon (12–1559)	2705 (46.7)	1204 (44.5)	1.09 (1.02-1.16)	1.08 (1.01-1.14)	
	Late pm (16–1830)	314 (5.4)	119 (37.9)	0.92 (0.78-1.06)	0.94 (0.80-1.07)	
Day of Survey	Mon-Thurs	3825 (66.0)	1581 (41.3)	1.0	1.0	<0.01
	Friday	931 (16.1)	378 (40.6)	0.97 (0.89-1.06)	0.97 (0.88-1.05)	
	Sat-Sun	1041 (18.0)	504 (48.4)	1.15 (1.06-1.24)	1.14 (1.05-1.23)	
Season of Survey	Rainy (Dec-Apr)	1566 (27.0)	694 (44.3)	1.0	1.0	0.20
	Cool, dry (May-Aug)	2646 (45.6)	1123 (42.5)	0.92 (0.78-1.05)	0.93 (0.81-1.05)	
	Cool, hot (Sept-Nov)	1586 (27.4)	646 (40.7)	0.89 (0.72-1.05)	0.87 (0.73-1.01)	

^a^Adjusted for household head, whether present all months in previous 6, household SEP and size, urban/rural residence and district; ^b^For adjusted PR and based on LRT

**Table 6:** Distribution of characteristics of eligible men by selection variables (*N* = 5797)

Characteristic	Detail	Monday-Thursday	Friday	Saturday-Sunday	*p*-value^a^
Household Head	No	1704 (65.8)	401 (15.5)	485 (18.7)	0.83
	Yes	2121 (66.1)	530 (16.5)	556 (17.3)	
Present continuously in previous 6mth	No	159 (71.3)	37 (16.6)	27 (12.1)	0.08
	Yes	3666 (65.8)	894 (16.0)	1014 (18.2)	
SEP Group	Lowest	603 (63.0)	157 (16.4)	197 (20.6)	<0.01
	Low	595 (57.3)	180 (17.3)	263 (25.3)	
	Middle	801 (66.4)	186 (15.4)	219 (18.2)	
	High	877 (68.8)	197 (15.5)	201 (15.8)	
	Highest	949 (71.8)	211 (16.0)	161 (12.2)	
Household Size	1-5	1817 (66.4)	438 (16.0)	481 (17.6)	0.49
	6–10	1758 (64.9)	449 (16.6)	504 (18.6)	
	>10	250 (71.4)	44 (12.6)	56 (16.0)	
Urban cluster	No	3104 (65.3)	750 (15.8)	897 (18.9)	0.43
	Yes	721 (68.9)	181 (17.3)	144 (13.8)	
District	Kafue	1507 (71.6)	287 (13.6)	312 (14.8)	0.09
	Chongwe	1792 (63.8)	464 (16.5)	554 (19.7)	
	Luangwa	526 (59.7)	180 (20.4)	175 (19.9)	
**Characteristic**	**Detail**	**Morning**	**Early pm**	**Late pm**	**p-value**
Household Head	No	1261 (48.7)	1183 (45.7)	146 (5.6)	0.28
	Yes	1517 (47.3)	1522 (47.5)	168 (5.2)	
Present continuously in previous 6mth	No	112 (50.2)	99 (44.4)	12 (5.4)	0.47
	Yes	266 (47.8)	2606 (46.8)	302 (5.4)	
SEP Group	Lowest	457 (47.8)	451 (47.1)	49 (5.1)	0.95
	Low	475 (45.7)	510 (49.1)	53 (5.1)	
	Middle	574 (47.6)	565 (46.9)	67 (5.6)	
	High	617 (48.4)	593 (46.5)	65 (5.1)	
	Highest	655 (50.0)	586 (44.4)	80 (6.1)	
Household Size	1-5	1288 (47.1)	1307 (47.8)	141 (5.2)	0.31
	6–10	1326 (48.9)	1247 (46.0)	138 (5.1)	
	>10	164 (46.9)	151 (43.14)	35 (10.0)	
Urban cluster	No	2279 (48.0)	2194 (46.2)	278 (5.9)	0.55
	Yes	499 (47.7)	511 (48.9)	36 (3.4)	
District	Kafue	993 (47.2)	980 (46.5)	133 (6.3)	0.42
	Chongwe	1352 (48.1)	1318 (46.9)	140 (5.0)	
	Luangwa	433 (49.2)	407 (46.2)	41 (4.7)	

^**a**^ *p*-value adjusted for clustering by study sites

## Abbreviations

HIV: Human Immunodeficiency Virus
HTC: HIV testing and counselling
ART: Anti-retroviral therapy
CRT: Cluster randomised trial
BHOMA: Better Health Outcomes through Mentoring and Assessment
PDAs: Personal digital assistant
DHS: Demographic and Health Surveys
SEP: Socioeconomic position
TB: Tuberculosis
PCA: Principal components analysis
PR: Prevalence ratio
adjPR: Adjusted prevalence ratio
LRT: Likelihood ratio test
ICC: Intra-cluster correlation
IQR: Interquartile range
